# Non-stoichiometric magnetite as catalyst for the photocatalytic degradation of phenol and 2,6-dibromo-4-methylphenol – a new approach in water treatment

**DOI:** 10.3762/bjnano.13.126

**Published:** 2022-12-15

**Authors:** Joanna Kisała, Anna Tomaszewska, Przemysław Kolek

**Affiliations:** 1 Department of Biology, Institute of Biology and Biotechnology, University of Rzeszow, Pigonia 1, PL-35-310 Rzeszow, Poland; 2 Department of Biotechnology, Institute of Biology and Biotechnology, University of Rzeszow, Pigonia 1, PL-35-310 Rzeszow, Poland; 3 Institute of Physics, University of Rzeszow, 1 Pigonia Street, PL-35-310, Rzeszów, Poland

**Keywords:** magnetite, ozonolysis, persistent organic pollutants, photocatalysis, water treatment

## Abstract

Phenol and 2,6-dibromo-4-methylphenol (DBMP) were removed from aqueous solutions by ozonolysis and photocatalysis. The properties and structural features of the catalysts and the organic compounds are discussed, as well as their influence on the degradation reaction rates. The degradation efficiency in photocatalytic processes was higher for DBMP (98%) than for phenol (approximately 50%). This proves the high efficiency of magnetite in the photocatalytic degradation of halogenated aromatic pollutants. The particularly high degradation efficiency regarding halogen-containing DBMP molecules and the yield of bromide ions indicate that DBMP degradation follows a mixed reduction–oxidation mechanism. DBMP molecules interact with the magnetite surface, enabling them to react with the available electrons, and, as a result, bromide ions can be released. The results confirm that magnetite is an effective photocatalyst in the degradation of halogenated aromatic pollutants.

## Introduction

Water is one of the most important natural resources on Earth. It helps to maintain environmental balance, but most of all, it is essential for human life and health. Although water covers more than 70% of our planet’s surface, only 2.5% is freshwater and less than 1% is accessible [1–2]. In the context of water consumption, there is an environmental indicator called the water footprint, which represents the sum of direct water consumption and virtual consumption (i.e., the amount of water needed to produce food and other consumer goods) [3]. Water consumption calculated in this way may amount to thousands of liters per person per year. Therefore, the use of water in a closed circuit is a major technological challenge. It is essential to design and develop new technologies for wastewater treatment and water recovery.

Bromophenols (BPs) are widely used as flame retardants (brominated flame retardants, BFRs), wood preservatives, and components in the polymer industry [4–5]. Bromophenols from various industries can cause severe contamination of soil, sediment, and water [6–7]. The United States Environmental Protection Agency (US EPA) has listed BPs as hazardous waste with strict environmental regulations to them [8]. Consequently, there is a growing need to develop processes for removing BPs from wastewater. In recent decades, much attention has been paid to advanced oxidation processes (AOPs) in the research and development of wastewater treatment technologies [7,9]. Processes such as cavitation, ozonation, Fenton chemistry, and photocatalysis have been successfully used to remove persistent organic pollutants (POPs) or as a pre-treatment in conventional or biological methods [10]. Ozonation is an AOP technique that has been widely used to remove organic compounds such as drugs, pesticides, petroleum constituents, and volatile organic compounds. Furthermore, the rate of ozonation is accelerated in alkaline media because hydroxide ions catalyze the decomposition of ozone and produce hyperactive hydroxyl radicals (^•^OH).

Photocatalysis is a promising technique for removing POPs from water using solar radiation as an energy source [11]. The photocatalysts are activated by radiation and produce highly reactive photo-induced charge carriers, which can react with the contaminants adsorbed on the surface of the catalyst. Understanding the properties of the photocatalyst material is critical to designing an effective photocatalytic process. The factors that influence photocatalytic efficiency include the photocatalyst bandwidth, the recombination rate of photogenerated electron–hole pairs, the use of solar energy, and problems with catalyst degradation.

Magnetite is a common auxiliary mineral in igneous and metamorphic rocks [12]. It is also found in sediments and soils. Magnetite has the smallest energy gap, the highest conductivity, and one of the lowest reduction potentials among natural minerals. It is an important reducer of heavy metals and organic pollutants in aquatic environments. Due to the mixed and variable valency of iron in its structure (Fe(III)_tet_[Fe(II),Fe(III)]_oct_O_4_), this oxide has unique properties [13–14]. At room temperature, magnetite is an inverse spinel conductor with Fe^3+^ on the tetrahedral sites and Fe^2+^ and Fe^3+^ on the octahedral sites. Electron hopping along the octahedral iron chain handles its conductivity and redox properties, causing the magnetite to initialize oxidation/reduction reactions. Fe_3_O_4_ nanoparticles have been used as a photocatalyst for the degradation of azo dyes [15], for wastewater treatment [16–17], for water decomposition, and for Cr(VI) reduction [18].

The study investigated the degradation of aqueous solutions of phenol (PhOH) and 2,6-dibromo-4-methylphenol (DBMP) via two processes, namely photocatalysis and ozonolysis. Two types of magnetite (M1 and M2) were used as catalysts in the photocatalysis process. The same type of magnetite catalysts has been investigated by us in our previous article [17] as photocatalysts for the degradation of 4,4′-isopropylidenebis(2,6-dibromophenol) in comparison with ozonolysis. Magnetite was chosen as a photocatalyst because of its low cost, interesting electron properties, and indisputably low environmental impact. The progress of the reaction was monitored by measuring the organic compound concentration. In order to determine the efficiency of the photocatalytic process, the organic compounds were also degradated through ozonolysis.

## Results and Discussion

**Figure 1:**
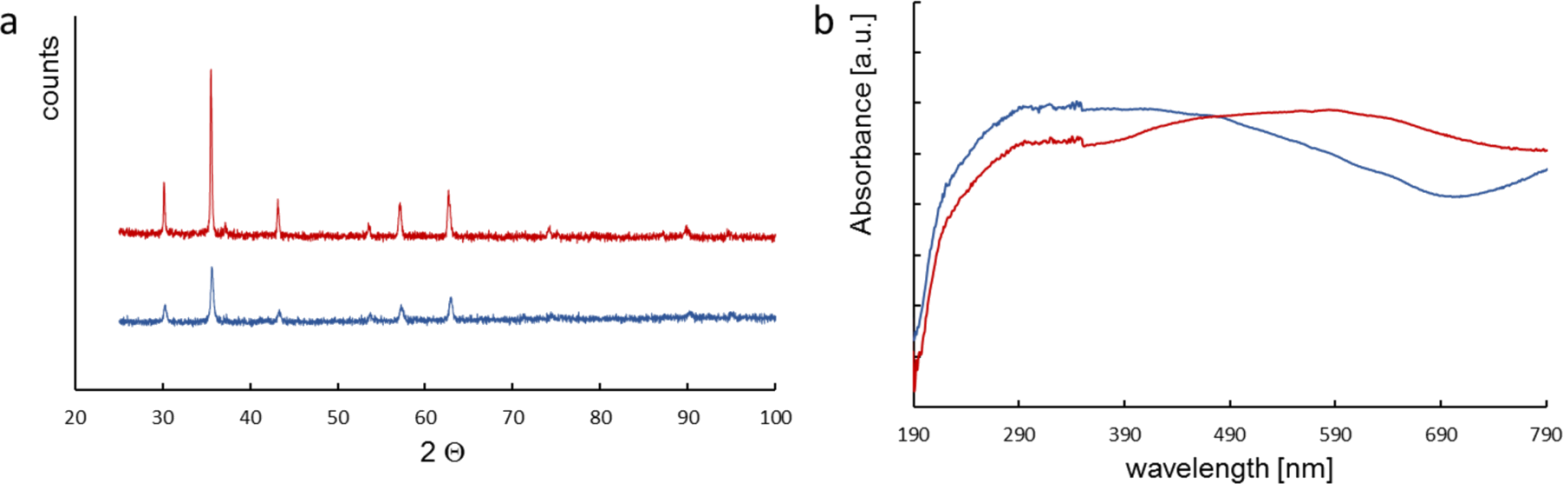
X-ray diffraction measurements of M1 (red), M2 (blue) (a), and UV–vis absorption spectra of M1 (red) and M2 (blue) (b). Figure 1a and 1b were adapted from [17] (© 2022 J. Kisała et al., distributed under the terms of the Creative Commons Attribution 4.0 International License, https://creativecommons.org/licenses/by/4.0).

The selected catalysts were characterized by SEM, X-ray diffraction, and ultraviolet–visible (UV–vis) analysis. The XRD and UV–vis results were published in our previous article [17]. We present this data again in this article as it is necessary for the discussion of the results. Zeta potential measurements were also presented in another previous publication of ours [19]. The absorption spectra of the catalysts showed noticeable differences (Figure 1b). Using the absorption spectra, the electron gap energies for M1 and M2 were determined to be 0.11 V and 1.75 V, respectively (Table 1) [20].

**Table 1:** Catalyst parameters.

Oxide	Size declared^a^ [nm]	Size measured^b^ [nm]	*a*^c^ [Å]	*x* ^d^	*E*_bg_ [eV]

M1	<5000	46	8.3845	0.4094	0.11
M2	<50	28	8.3595	0.1829	1.75

^a^Size declared by the vendor; ^b^XRD measurements; ^c^length of the edge of the magnetite unit cell; ^d^*x* = Fe^2+^/Fe^3+^.

Phase identification of the magnetite structure was performed using a powder X-ray diffractometer (Figure 1a). The XRD spectra revealed that the diffraction peaks at 2θ = 30.2°, 35.3°, 43.7°, 53.9°, 57.1°, and 62.7° (Figure 1a) correspond to those of Fe_3_O_4_ (reference code COD 01-089-3854); they belong to a cubic structure system corresponding to the facets (220), (311), (400), (422), (511), and (440) of Fe_3_O_4_, respectively [21]. The absence of the (210) and (211) peaks confirms that the catalysts were indeed magnetite. The mean size of the catalyst crystallites (*D*) was calculated from the high-reflection X-ray diffraction profiles by measuring the full width at half maximum (FWHM), using the Scherrer equation (Equation 1) [22–23]:


[1] $$D=\frac{0.89\lambda }{B\cos\theta },$$

where λ is the X-ray wavelength in nanometers, *B* is the width at half peak-height in radians, and θ is the angle between the incident and diffracted beams in angular degrees.

Magnetite oxidation (determined as the parameter *x* = Fe^2+^/Fe^3+^) can range from 0.5 (stoichiometric magnetite Fe(III)_tet_[Fe(II),Fe(III)]_oct_O_4_) to 0 (completely oxidized; maghemite, γ-Fe_2_O_3_) [24]. A magnetite with *x* values in the range of 0 < *x* < 0.5 is defined as non-stoichiometric or partially oxidized magnetite. Stoichiometric magnetite and maghemite are significantly different. Magnetite is a conductor (bandgap of 0.1 eV), while maghemite is a semiconductor (bandgap of approx. 2.0 eV) [12]. The unit cell parameter of magnetite is slightly larger (ca. 8.40 Å) than that of maghemite (ca. 8.34 Å). A combination of these properties is what characterizes non-stoichiometric magnetite. The length of the edge of the magnetite unit cell is linearly related to the stoichiometry (for *x* = 0, *a* = 8.3390 Å; for *x* = 0.25, *a* = 8.3662 Å; for *x* = 0.5, *a* = 8.3942 Å) [25]. Knowing the cell length from the XRD measurements makes it possible to determine *x* for the catalysts under study (Table 1). The determined *x*-values indicate that the catalysts were non-stoichiometric magnetites. M1 with a larger grain diameter is less oxidized while M2 is highly oxidized. This is also reflected in the electron bandgap energy. These values show that the tested catalysts were semiconductors rather than conductors.

**Figure 2:**
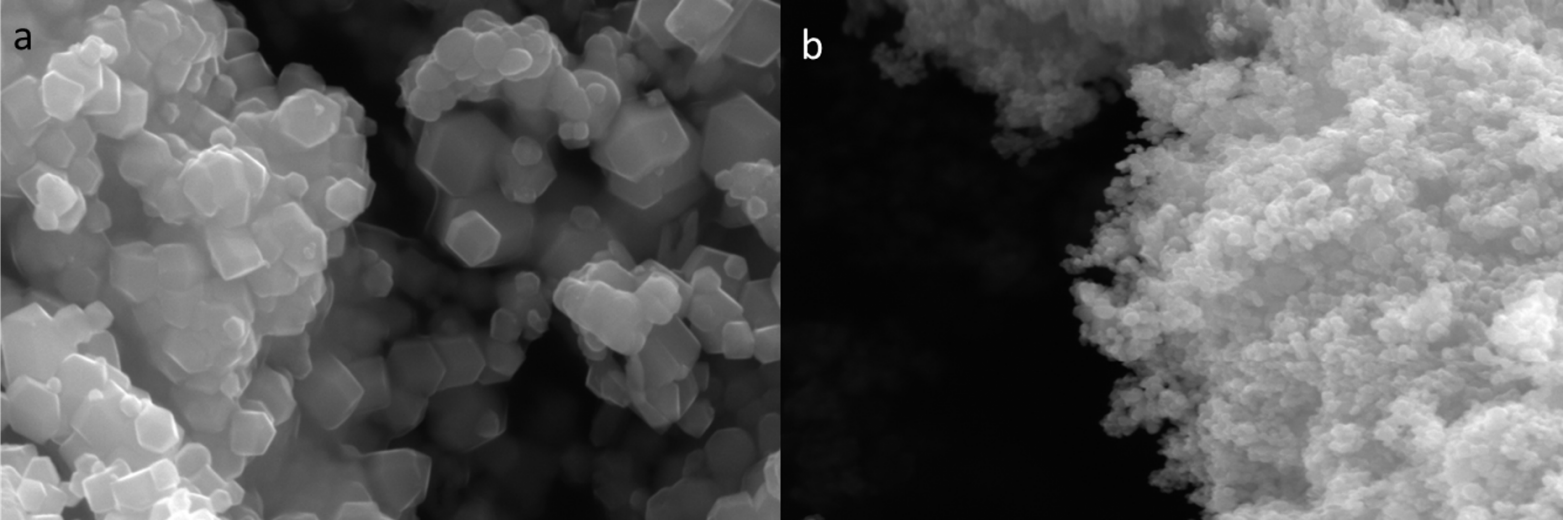
SEM images of M1 (a), and M2 (b) catalysts.

The morphology of the Fe_3_O_4_ catalysts is shown in Figure 2. The images show nanocrystal agglomerates with particle sizes of 100–400 nm for M1 and of 25–100 nm for M2 (Figure 2). The aggregation of M2 particles is much stronger than that of M1. Hence, M2 forms a porous structure.

There is a disparity in the particle sizes measured with SEM and XRD, because the particle sizes detected using these two techniques are not in the same orientation, and the terms "particle size" and "crystal size" refer to different concepts (a particle may contain several or only one crystallite) [26]. The particle size can be calculated from the XRD measurements using the Scherrer equation (Equation 1). The sizes of the crystal structures are determined by analyzing the diffraction intensity of the X-ray beams. The lines in a powder diffraction pattern have a finite width, but the lines are wider than usual if the particles are small (widening decreases as the particle size increases). Line broadening is used to estimate the mean particle size, but peak broadening may have many causes other than crystal size. Widening of the diffraction peaks can result from both instrument constraints and the sample (all types of crystal imperfections would lead to an additional widening of the XRD peak due to microstrain, resulting in a lower apparent crystal size value). This means that the Scherrer equation is an extremely rough estimate of the minimal size of crystals whereas SEM reveals the maximum size of the particles.

The catalytic activity of commercially available M1 and M2 was evaluated through the photocatalytic degradation of phenol and DBMP. The photocatalytic activity was compared with the efficiency of ozonolysis. The photocatalytic efficiency is improved by the adsorption of organic compounds onto the surface of the catalyst. The sorption power depends largely on the properties of the organic molecule in question, as well as on the properties of the catalyst’s surface. The test reactions were carried out in aqueous solution at pH 8 (due to the hydrolytic stability of the catalysts). Under acidic conditions, magnetite dissolves according to Equation 2:


[2] $${\text{Fe}}_{3}{\text{O}}_{4}+2{\text{H}}^{+}\to {\text{Fe}}^{2+}+\gamma {\text{-Fe}}_{2}{\text{O}}_{3}+{\text{H}}_{2}\text{O.}$$

**Figure 3:**
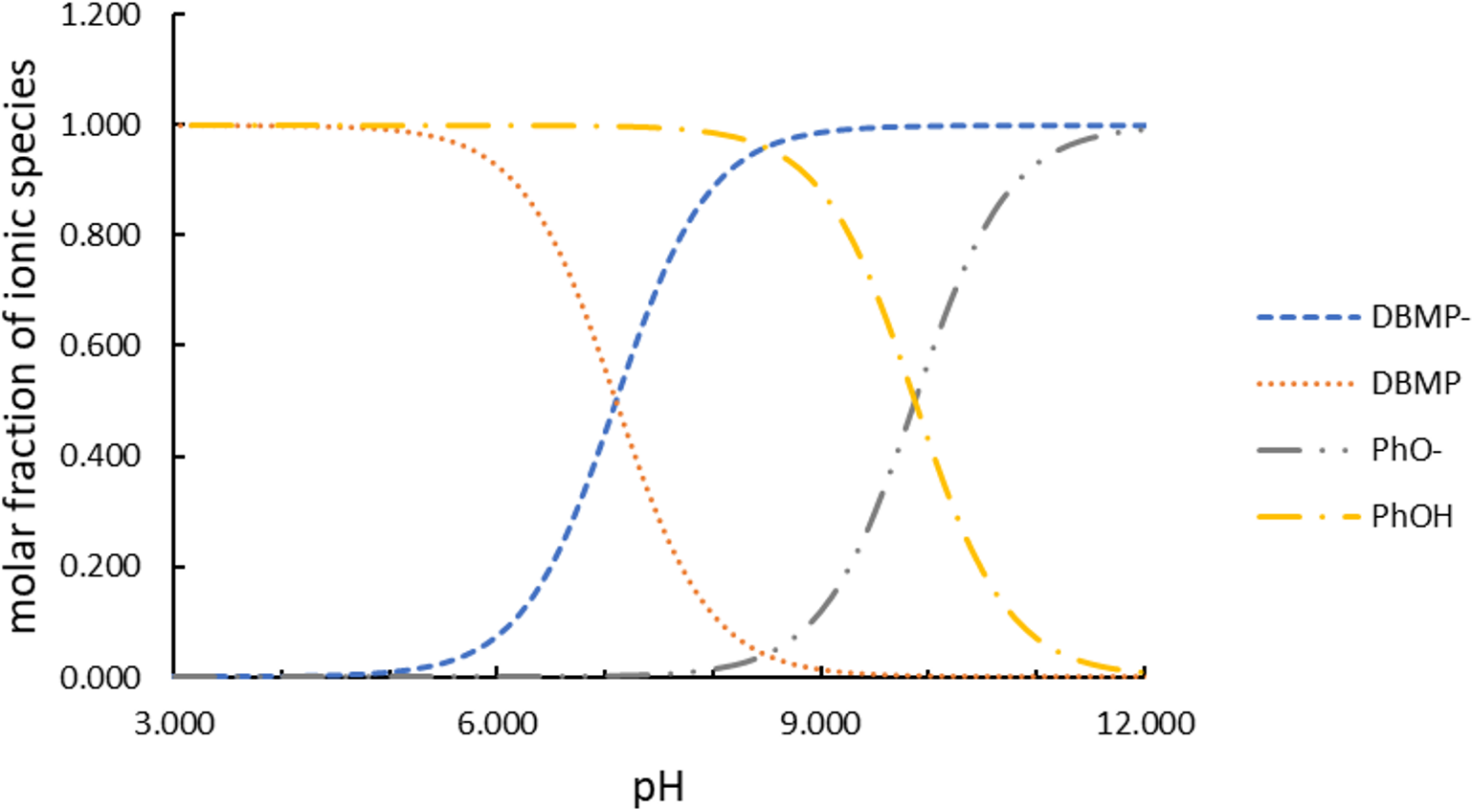
Molar fractions of ionic species as functions of the pH value.

At pH > 7, however, the effect of hydrolysis is expected to be negligible [27]. The molar fractions of the ionic forms of organic compounds present in the solution at pH 8 were calculated using the software CurTiPot [28] based on the published p*K*_a_ values for solutes (Figure 3). The resulting values were as follows: DBMP (0.112); DBMP^−^ (0.888); PhOH (0.987); and PhO^−^ (0.013).

The properties of the catalyst’s surface are essential in photocatalysis. Adsorption of organic compounds on the surface of the catalyst is strongly affected by the surface charge. The point of zero charge (PZC) is defined as the pH value at which the catalyst exhibits a net zero surface charge. At pH values below the PZC, the surface of the catalyst is protonated and has a positive net surface charge. In turn, the net surface charge becomes negative at pH values above the PZC.

**Figure 4:**
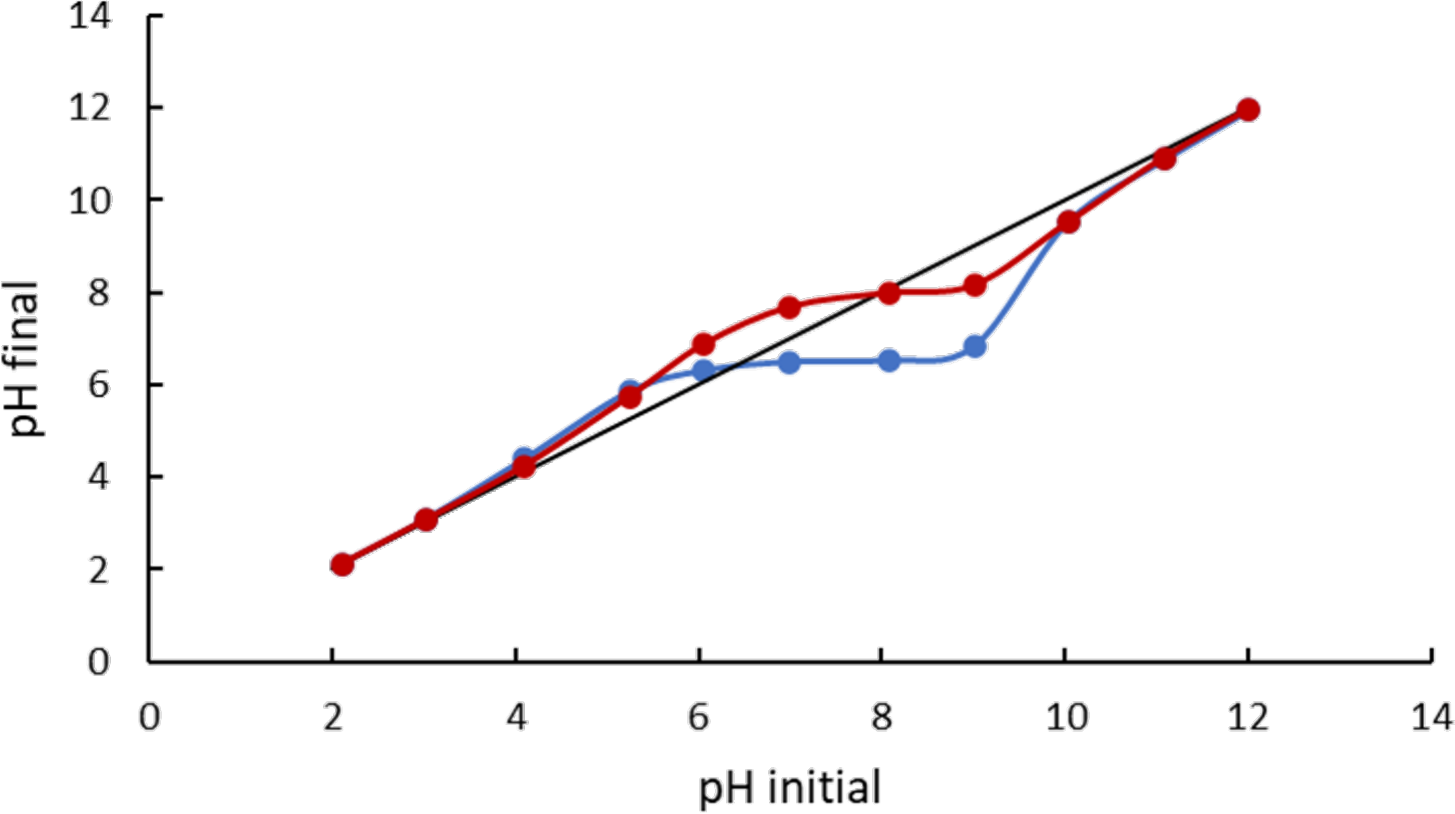
Determination of pH_PZC_ for M1 (red) and M2 (blue).

As expected, the curves of the final pH as a function of the initial pH of the catalysts in 0.1 mol·L^−1^ NaCl show an intersection with the line *y* = *x*, which corresponds to the PZC of the sample (Figure 4). The measurement of the PZC exhibits a higher pH_PZC_ value for M1 (8.0) than for M2 (6.2) (Figure 4). The differences in these pH_PZC_ values may result from the oxidation levels of M1 and M2, the latter having a positively charged surface because it is more oxidized and the former being negatively charged due to a lower oxidation level (see the *x* value, in Table 1). As a result, at pH 8 the surfaces of these two magnetite catalysts have opposite charges, which was shown in the zeta potential measurements of the catalysts (−0.37 mV and +14.4 mV for M1 and M2, respectively). These findings are in line with those previously observed by Hou et al. [29] on analogous samples.

It is believed that, at the catalyst–solution interface, the phenoxy group binds specifically to surface sites (Equations 3 and 4):


[3] $$\equiv \text{Fe}\left(\text{II,III}\right)\text{OH}+{\text{S}}^{n-}\to \;\equiv \text{Fe}\left(\text{II,III}\right)\text{OH}\cdot \cdot \cdot {\text{S}}^{n-}$$

[4] $$\equiv \text{Fe}\left(\text{II,III}\right){\text{O}}^{-}+\text{S}\to \;\equiv \text{Fe}\left(\text{II,III}\right){\text{O}}^{-}\cdot \cdot \cdot \text{S}$$

S – undissociated organic compound; S*^n^*^−^ - dissociated organic compound

This means that DBMP is more likely to interact with the catalyst surface than PhOH. The distribution of species shows that only approximately 0.013 molecules of phenol will react with the protonated catalyst surface, while 0.888 molecules of DBMP could interact with the catalyst surface by Coulombic forces and a further 0.112 via hydrogen bonds.

The apparent degradation rate constant (*k*_app_) was determined (for each degradation system) according to Equation 5, assuming that the reactions occurring were of pseudo-first order.


**Figure 5:**
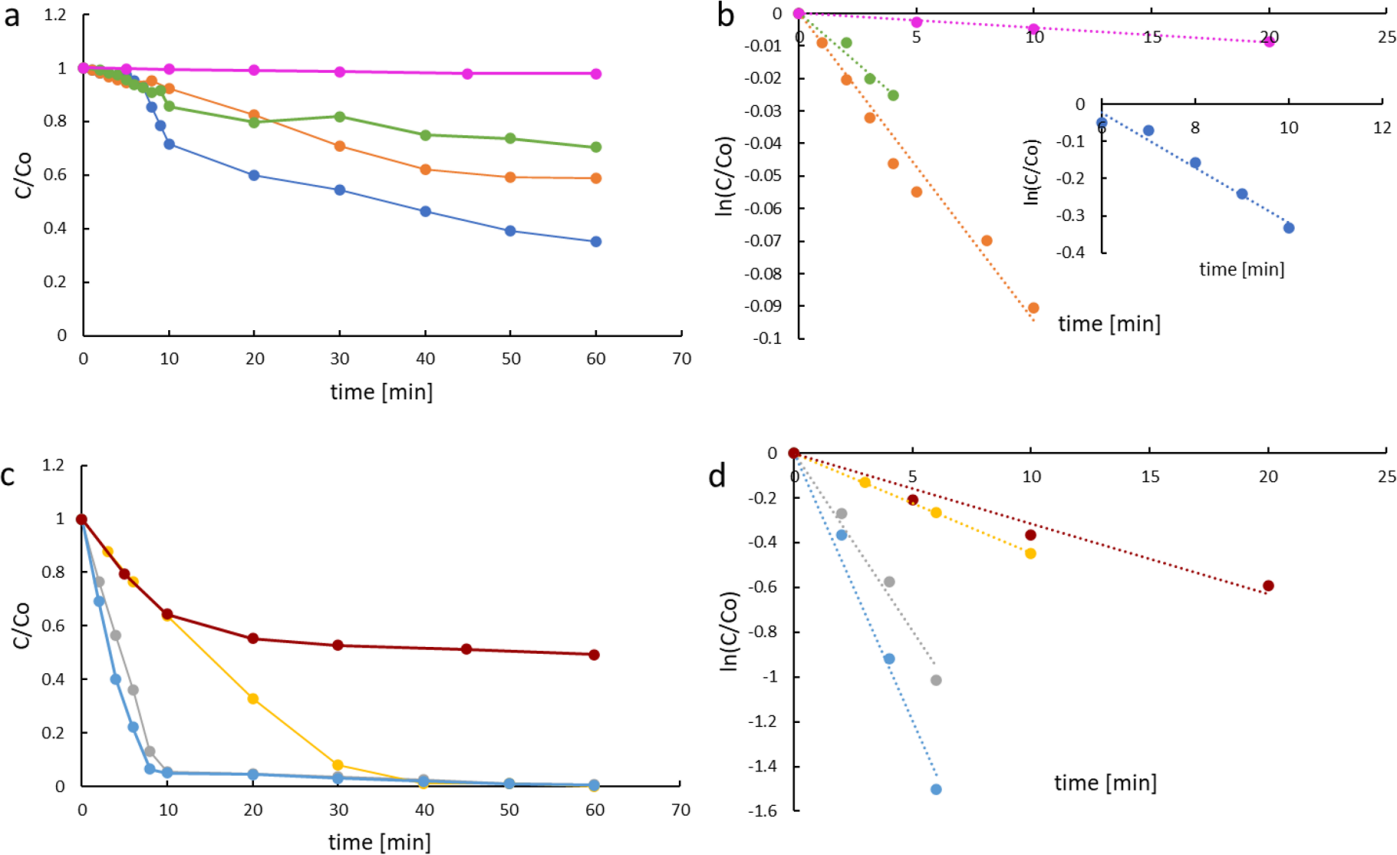
(a) Substrate decay rate of PhOH/O_3_ (blue), PhOH/M1 (orange), PhOH/M2 (green), and PhOH/photolysis (pink); (b) plot of ln(C*_t_*/C_0_) vs irradiation time for phenol; (c) substrate decay rate of DBMP/O_3_ (yellow), DBMP/M1 (grey), DBMP/M2 (light blue), and DBMP/photolysis (red); (d) plot of ln(C*_t_*/C_0_) vs irradiation time for DBMP.

[5] $$\ln\left(C_{t}/C_{0}\right)=-k_{\text{app}}\;t,$$

where *C*_0_ and *C_t_* are the initial concentration and the concentration at time *t*, respectively. The dependence of ln(*C_t_*/*C*_0_) on time is represented by straight lines, as shown in Figure 5b,d. Therefore, the degradation kinetics are consistent with the pseudo-first-order kinetic model (*R*^2^ > 0.95). The results demonstrate that the photocatalytic processes were very efficient and more efficient than degradation by ozonolysis. The values of the degradation rate constants and the half-lives are summarized in Table 2. Significant differences in reactivity were observed for phenol and DBMP. The dissociation constant (p*K*_a_) of DBMP is approx. 7.21, hence, almost 90% of the DBMP was dissociated at pH 8, while phenol was mainly (98%) undissociated (Figure 3). The high amount of the ionic form results in rapid direct photolysis. Direct photolysis of aqueous DBMP was mainly initiated by photolytic cleavage of the bromine–carbon bond and the formation of bromide ions. The half-lives of direct photolysis for phenol and DBMP were 1732.9 and 22 min, respectively. These results indicate that the bromine substituent facilitated the direct photolysis of the phenols. The apparent rate constant of DBMP degradation during direct photolysis and through photocatalysis were, respectively, 31.5 × 10^−3^ min^−1^ and 149 × 10^−3^ min^−1^ for M1 and 220 × 10^−3^ min^−1^ for M2. As a result, approximately 50% and 98% of DBMP was degraded via direct photolysis and photocatalysis, respectively (after 60 min). The efficiency of phenol photocatalysis was low (ca. 40% for M1 and ca. 30% for M2, after 60 min). This may be due to the lack of interaction between the catalysts’ surfaces and phenol.

**Table 2:** Kinetic parameters of the reaction systems under study.

Reaction system	*k*_app_ [min^−1^]	*t*_1/2_ [min]	R^2^	*k*_app_ [min^−1^] bromide formation	R^2^

PhOH/O_3_	73.6 × 10^−3^	15.4	0.97	—	—
PhOH/M1	11.0 × 10^−3^	63.0	0.99	—	—
PhOH/M2	6.2 × 10^−3^	111.8	0.97	—	—
PhOH/photolysis	4.0 × 10^−4^	1732.9	0.99	—	—
DBMP/O_3_	44.6 × 10^−3^	15.5	0.99	197.3 × 10^−3^	0.83
DBMP/M1	159.7 × 10^−3^	4.3	0.98	354.2 × 10^−3^	0.99
DBMP/M2	239.7 × 10^−3^	2.9	0.99	409.7 × 10^−3^	0.91
DBMP/photolysis	31.5 × 10^−3^	22.0	0.96	50.7 × 10^−3^	0.99

The hydroxyl radicals (^•^OH) generated during water ozonation are described by the SBH model [30–32] for neutral pH or the TFG model [33–35] for pH > 7. Considering the process conditions during the study (pH 8), we are interested in the TFG model. The rate constants used in the model were determined by Chelkowska et al. [36]. The TFG model includes the following reactions:


[6] $${\text{O}}_{3}+{\text{OH}}^{-}\to {\text{HO}}_{2}{}^{-}+{\text{O}}_{2}\;k_{6}=120\;{\text{M}}^{-1}\;{\text{s}}^{-1}$$

[7] $${\text{HO}}_{2}{}^{-}+{\text{O}}_{3}\to {\text{O}}_{2}{}^{\cdot -}+{\text{HO}}^{\cdot }+{\text{O}}_{2}\;k_{7}=1.5\;\times \;10^{6}\;{\text{M}}^{-1}\;{\text{s}}^{-1}$$

[8] O2⋅−+O3→O3⋅−+O⋅2             k8=1.6 × 109 M−1 s−1

[9] O3⋅−+H2O→O⋅H+OH−+O2  k9=15 M−1 s−1

The reaction in Equation 6 shows that the ozone decomposition process is initiated by hydroxy anions. Two-electron transfer of the oxygen atom produces the ^–^OOH anion, which is necessary for the generation of hydroxyl radicals. The low value of the reaction rate constant (Equation 6) indicates that it is a limiting process of the phenol ozonolysis, hence the observed delay in the rate of phenol degradation in ozonolysis in Figure 5a,b. Because the reaction in Equation 6 is slow, the reaction conditions are not stationary at the initial stage of the ozonolysis process. After reaching static conditions (about 6 min from the start of gas purging), the process proceeds according to the pseudo-first-order kinetic model. The determination of the apparent rate constant of phenol ozonolysis after obtaining steady-state process conditions is shown in the insert in Figure 5b.

The degradation of phenol at pH 8 in both ozonolysis and photocatalysis is oxidative (reaction with hydroxyl, superoxide, and hydroperoxide radicals). When comparing the apparent degradation rate constants (*k*_app_) of phenol for ozonolysis (73.6 × 10^−3^ min^−1^) and for photocatalysis (11 × 10^−3^ min^−1^ for M1 and 6.2 × 10^−3^ min^−1^ for M2), a higher *k*_app_ value can be observed for ozonolysis.

**Figure 6:**
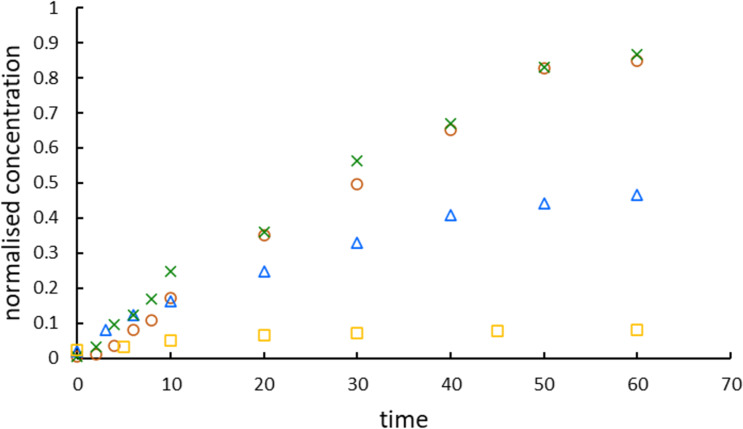
Bromide ion production as a function of the time (circles – M1, crosses – M2, triangles – ozonation, squares – photolysis).

**Figure 7:**
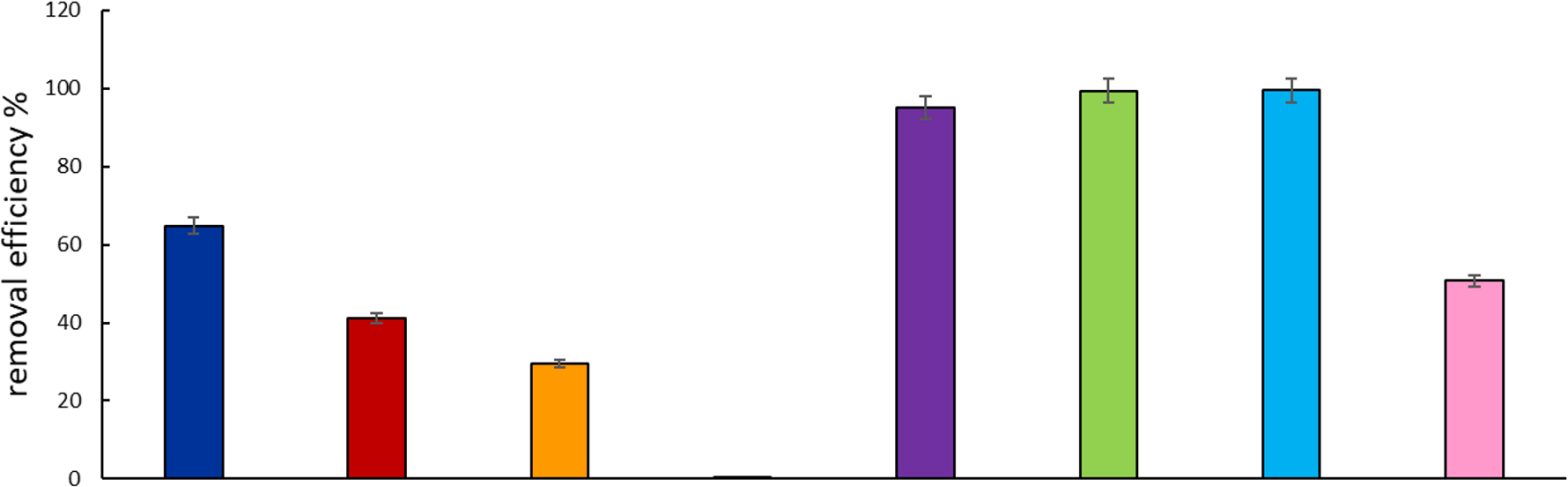
Substrate removal efficiency of PhOH/O_3_ (dark blue), PhOH/M1 (red), PhOH/M2 (orange), PhOH/photolysis (black), DBMP/O_3_ (violet), DBMP/M1 (green), DBMP/M2 (light blue), and DBMP/photolysis (pink).

As shown in Figure 6, bromide ions were released from the benzene ring by the ozonation reaction. The bromide ion concentration increased with an increasing level of degradation of DBMP, which suggests that degradation generates brominated intermediates that are subsequently decomposed to release bromine atoms. The normalized concentration of bromide ions (with DBMP degradation efficiency close to 95%; Figure 7) is about 0.9 (where 1 is the theoretical complete release of bromide; Figure 6). However, a normalized bromide ion concentration smaller than 0.5 for ozonolysis may denote the production of brominated organic by-products. Release yields of bromine in the form of bromide ions as high as 90% showed eventual mineralization. The half-live of DBMP in photocatalytic processes was 4.6 min (M1) or 3.2 min (M2); for ozonolysis, it was 15.5 min, suggesting a faster degradation of DMBP in photocatalytic processes than in ozonolysis. The apparent rate constants of bromide generation for all processes in the study are summarized in Table 2. The results are consistent with those for degradation. The degradation activity of DBMP was in the order of M2 > M1 > O_3_ > photolysis, and the same order was observed for the generation of bromide anions (Figure S2, Supporting Information File 1). The higher yields of bromides in the case of photocatalysis revealed the contribution of the reduction process to the photocatalytic degradation of DBMP.

Figure 7 shows the degradation efficiency regarding phenol and DBMP. Phenol degradation reached approx. 65% after 60 min of ozonation whereas the degradation of DBMP was 95%. This is related to the ionic forms of these compounds under the given reaction conditions (89% of DBMP was dissociated vs only 13% of phenol). The presence of hydroxyl radicals in the solution and a higher reactivity of phenoxide ions compared to that of undissociated forms contributed to a higher degradation efficiency of DBMP in ozonolysis. DBMP is easier to oxidize than phenol. This is also because of the substitution of two hydrogen atoms by two electron donors (–Br) in the DBMP molecule, which activates the aromatic ring. The degradation efficiency in photocatalysis was higher for DBMP than for phenol, reaching 98%.

Excitation of the catalyst with energies higher than the bandgap energy generates holes and electrons, which, after moving to the catalyst surface, may participate in redox processes. In a basic medium, the photocatalytic process may proceed by oxygen reduction at the surface of the particles (electron transfer only) [37]. A similar electron transfer can occur during the adsorption of organic compounds on magnetite. In the presence of adsorbed aryl halogenated compounds on the catalyst surface, the accumulated electrons are available to activate carbon–halogen bonds via dissociative electron transfer [38–39]. The electron from the catalyst conduction band is injected into the unoccupied orbital of halogenated aromatics, resulting in the breaking of carbon–halogen bonds.

For the Fe^2+^ and Fe^3+^ ions on the octahedral sites, electron transfer between these ions is feasible without substantial excess energy [40]. Therefore, electrons could be transferred with very low activation energy among iron ions on the octahedral sites. As we showed above, DBMP interacts with the magnetite surface, thus, it can react with the generated electrons. The following reaction path for DBMP degradation is proposed to explain our observations (a detailed description of DBMP reactions can be found in Figure S3, Supporting Information File 1):


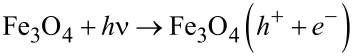



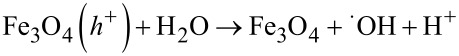



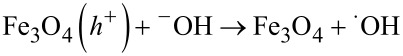



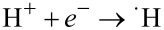



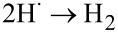



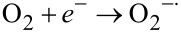



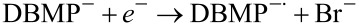



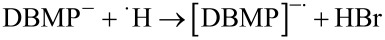



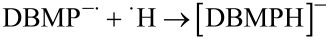



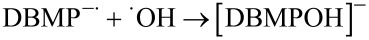



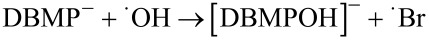



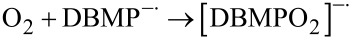



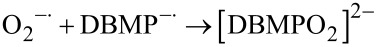


The results demonstrate the effectiveness of magnetite in the photocatalytic degradation of halogenated aromatic pollutants.

## Conclusion

In the present study, two types of magnetite (M1 and M2) were investigated as possible photocatalysts. The morphology, optical properties, and structural properties of the M1 and M2 samples were investigated. The stoichiometry of magnetite noticeably impacted the optical and surface properties.

Direct photolysis indicated that the bromine substituent facilitated the degradation of phenols. In the ozonation process, the degradation of phenol reached approximately 65% after 60 min, while the degradation of the DBMP reached 95% after the same time. The kinetic results showed that photocatalytic processes were more effective than DBMP degradation by ozonolysis. Phenol degradation occurs mainly via oxidation, as indicated by comparable rate constants for all the degradation processes studied. In the case of photocatalysis, the degradation rate increases with increasing values of the catalyst’s zeta potential. This indicates that the apparent rate constant values are influenced by the interaction between the DBMP molecule and the magnetite surface. When bonded to the catalyst’s surface, molecules can react with the electrons being generated. Therefore, we postulate a mixed reductive–oxidative mechanism of DBMP degradation on magnetite.

In conclusion, magnetite can be an efficient and low-cost material suitable for removing halogenated aryl compounds from aqueous solutions.

## Experimental

### Materials

The 2,6-dibromo-4-methylphenol (97%) was purchased from Alfa Aesar, phenol (>99%) was obtained from Riedel-de-Haen. The magnetite samples were obtained from Sigma-Aldrich: Fe_3_O_4_ micro-powder (M1, particle size under 5 µm, *d* = 4.8–5.1 g·cm^−3^, 95% purity estimated from trace metal analysis); Fe_3_O_4_ nano-powder (M2, particle size under 50 nm, *d* = 3.9 g·cm^−3^, >98% purity estimated from trace metal analysis). All chemicals were of analytical grade and were used without prior purification.

### Characterization of the photocatalysts

The PZC of catalysts was measured according to Kocharova and co-workers [41]. For the determination of pH_PZC_, eleven vials were filled with 0.1 mol·L^−1^ NaCl solution. The pH of each vial was adjusted with NaOH and HCl solutions to pH 2–12. Next, 10 mg of catalyst was dispersed in each vial. The solution was constantly agitated at 240 rpm for 3 h at ambient temperature to reach equilibrium. The equilibrium pH value was measured with a multimeter (CPC 411, Elmetron, Poland) and the values were plotted against the initial pH values for both series. The PZC value was then obtained from the point at which the curve showing final pH vs initial pH intersected the *y* = *x* line on the graph. The morphology of the catalysts was observed with a field-emission scanning electron microscope (Zeiss Ultra 55, Oberkochen, Germany). The crystalline phases were analyzed with a Cu Kα powder diffractometer (D8 Advance, Bruker, Ettlingen, Germany) operating at 40 kV and 36 mA (λ = 0.154056 nm). The optical characterization of the catalysts was performed by using a spectrophotometer (Cary Series UV-Vis-NIR, Agilent Technologies) in the wavelength range of 190–800 nm.

### Photocatalytic degradation of phenols

The photocatalytic activity of M1 and M2 was evaluated by the photocatalytic degradation of PhOH and DBMP. First, 750 cm^3^ (1.064 × 10^−3^ mol·L^−1^) of aqueous phenol solution was placed into the reactor. The pH of the solution was adjusted to 8 ± 0.1 with 0.1 mol·L^−1^ NaOH. Then, 0.67 g·L^−1^ of the catalyst powder was dispersed in the phenol solution. The resulting suspension was stirred for 30 min in the dark (until adsorption/desorption equilibrium was reached). Photocatalytic degradation was carried out using a glass photoreactor (Heraeus LRS2, Hanau, Germany) in air. The irradiation was performed with a TQ150 excimer lamp (150 W, with forced water cooling to 25 °C, 47 W light energy flux of power density 4.7 mW·cm^−2^ measured by a Peak Tech digital lux meter) immersed in the continuously stirred reaction suspension. The photocatalytic reaction was performed for 60 min. During the reaction, 2 mL samples were collected from the reactor at regular time intervals (from the first to the tenth minute, every 2 min, then every 10 min up to 1 h), then filtered through a 0.22 μm syringe filter. The organic compound concentrations were evaluated by using HPLC.

### Ozonolysis of phenols

For the ozonolysis experiment , the aqueous phenol solution (1.064 × 10^−3^ mol·L^−1^, 750 cm^3^, pH 8 ± 0.1, adjusted with 0.1 mol·L^−1^ NaOH) was continuously purged with gas (ozone solution in air). The ozonolysis reaction was performed using an ozone generator (Viaken Vairo-2186, Krakow, Poland). The ozone concentration in the gas stream was determined by the iodometric method [42] to be 12.73 mg·L^−1^·h^−1^. The ozonolysis reaction was performed for 60 min, during which samples were collected from the reactor at regular time intervals (from the first to the tenth minute, every 3 min, then every 10 min up to 1h). The organic compound concentrations were evaluated by using HPLC.

### Analysis

Changes in phenol concentration were determined by a high-performance liquid chromatography system (Shimadzu, Japan) equipped with a UV detector (SPD-10AV) and a C18 column (Knauer 250 × 4.6 mm, Eurospher II 100-5 C18 H, with precolumn). The analysis conditions were as follows: mobile phase: 70% acetonitrile and 30% water; flow rate: 1.0 cm^3^·min^−1^; injection volume: 20 × 10^−3^ cm^3^; absorbance detection: 270 and 310 nm for PhOH and DBMP, respectively. External standards of seven concentration levels ranging from 1 × 10^−4^ to 1 × 10^−2^ mol·L^−1^ were used to quantify PhOH and DBMP.

The concentration of dissolved bromide ions was determined potentiometrically with a bromide ion-selective electrode (EBr-01, Hydromet, Poland) with a silver chloride electrode (RL-100, Hydromet, Poland) as a reference electrode and a multimeter (CPC 411, Elmetron, Poland). External standards of seven concentration levels ranging from 1 × 10^−5^ to 1 × 10^−3^ mol·L^−1^ were used to quantify bromide ions.

## Supporting Information

Figure S1: Kubelka–Munk plots of M1 (red) and M2 (blue); Figure S2: Establishing the apparent rate constant for bromide generation; Figure S3: DBMP reactions with reactive species generated during photocatalysis.

File 1 Supplementary information.
