# Supplementary material for: Non-stoichiometric magnetite as catalyst for the photocatalytic degradation of phenol and 2,6-dibromo-4-methylphenol – a new approach in water treatment
Source: Beilstein J Nanotechnol. 2022 Dec 15;13:1531–40. doi: 10.3762/bjnano.13.126 (PMC9764857; doi:10.3762/bjnano.13.126)
Supplement: File 1 — Supplementary information. [file Beilstein_J_Nanotechnol-13-1531-s001.pdf]

BEILSTEIN JOURNAL OF NANOTECHNOLOGY

Supporting Information

for

Non-stoichiometric magnetite as catalyst for the photocatalytic degradation of phenol and 2,6-dibromo-4-methylphenol – a new approach in water treatment

Joanna Kisała, Anna Tomaszewska and Przemysław Kolek

Beilstein J. Nanotechnol. **2022,** *13,* 1531–1540. doi:10.3762/bjnano.13.126

Supplementary information

License and Terms: This is a supporting information file under the terms of the Creative Commons Attribution License (https://creativecommons.org/licenses/by/4.0). Please note that the reuse, redistribution and reproduction in particular requires that the author(s) and source are credited and that individual graphics may be subject to special legal provisions.
The license is subject to the *Beilstein Journal of Nanotechnology* terms and conditions: (https://www.beilstein-journals.org/bjnano/terms)

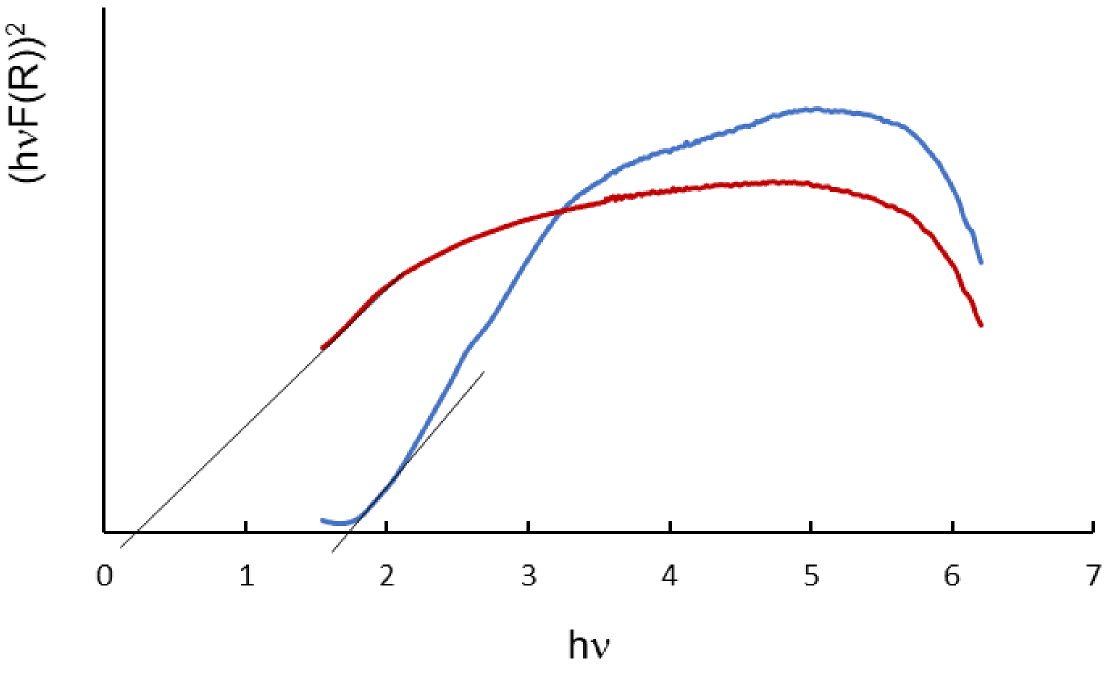

Figure S1: Kubelka–Munk plots of M1 (red) and M2 (blue).

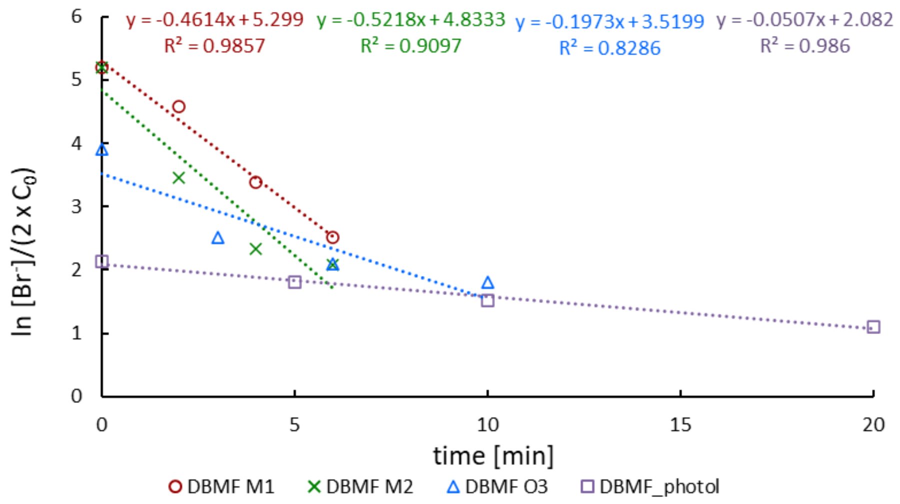

Figure S2: Establishing the apparent rate constant for bromide generation.

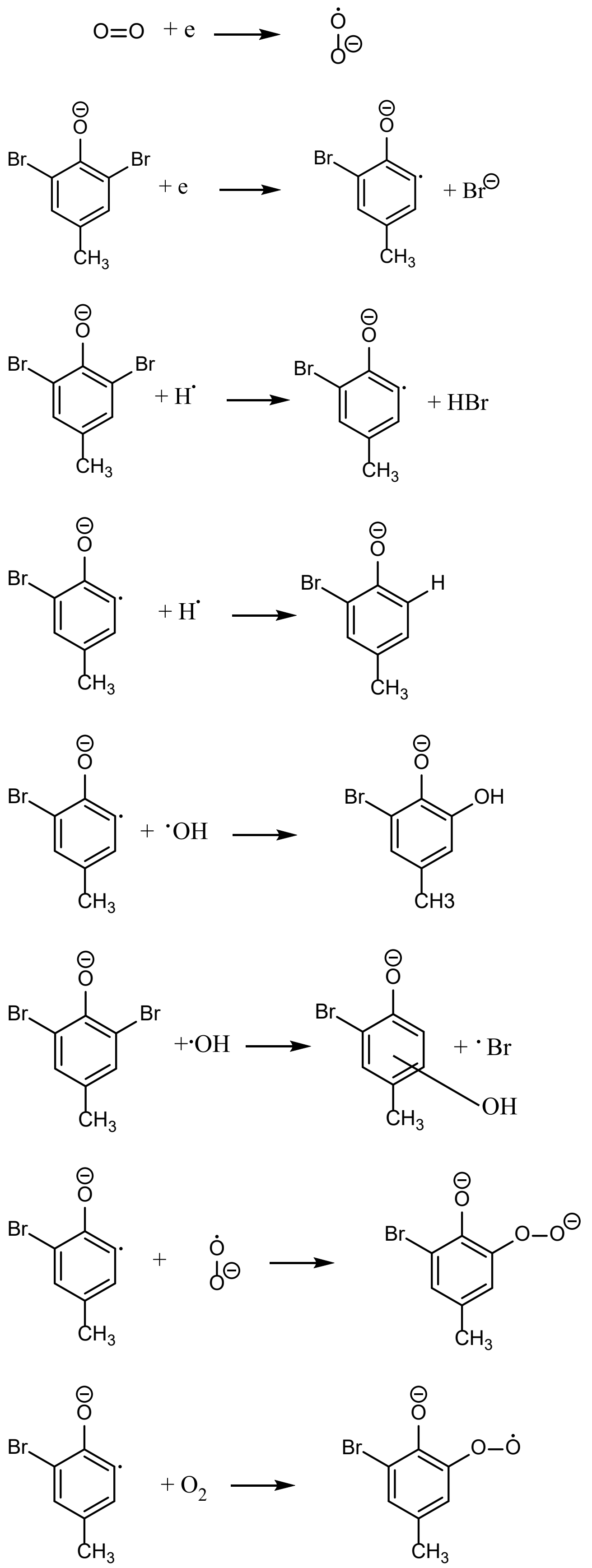

Figure S3: DBMP reactions with reactive species generated during photocatalysis.
